# Food and Development: Children and Adolescents with Neurodevelopmental and Comorbid Eating Disorders—A Case Series

**DOI:** 10.3390/bs13060499

**Published:** 2023-06-13

**Authors:** Jacopo Pruccoli, Giulia Guardi, Angela La Tempa, Beatrice Valeriani, Francesca Chiavarino, Antonia Parmeggiani

**Affiliations:** 1 Centro Regionale per i Disturbi della Nutrizione e dell’Alimentazione in età Evolutiva, IRCCS Istituto delle Scienze Neurologiche di Bologna, U.O. Neuropsichiatria dell’età Pediatrica, 40138 Bologna, Italy; jacopo.pruccoli2@unibo.it (J.P.); giulia.guardi@studio.unibo.it (G.G.); angela.latempa@studio.unibo.it (A.L.T.); francesca.chiavarino@studio.unibo.it (F.C.); 2 Dipartimento di Scienze Mediche e Chirurgiche (DIMEC), Università di Bologna, 40126 Bologna, Italy; 3 Centre for Chronic Intestinal Failure—Clinical Nutrition and Metabolism Unit, Universitaria di Bologna, IRCCS Azienda Ospedaliero, 40138 Bologna, Italy; beatrice.valeriani@aosp.bo.it

**Keywords:** neurodevelopmental disorders, autism spectrum disorder, attention-deficit/hyperactivity disorder, specific learning disorders, anorexia nervosa, bulimia nervosa, binge eating disorder, avoidant/restrictive food intake disorder

## Abstract

The impact of psychiatric comorbidities in the diagnosis and treatment of feeding and eating disorders (FEDs) represents an emerging research topic. The current literature, nonetheless, lacks studies investigating the developmental paths of individuals with FEDs and comorbid neurodevelopmental disorders (NDDs). Here, we report 11 cases of children and adolescents with comorbid FEDs and NDDs, as assessed along the neuropsychological, psychopathological, and nutritional developmental pathways. The onset of FED-related psychopathology was preceded, sometimes undiagnosed, by altered neurodevelopmental features leading to specific NDD diagnoses (autism spectrum disorder—ASD; attention-deficit/hyperactivity disorder—ADHD; specific learning disorder—SLD). NDDs appeared to influence the diagnoses and treatments of FEDs, frequently with an impact on socio-relational and emotional premorbid features, and on the possibility to receive and attend FED-targeted treatments. Further studies should longitudinally contribute to assessing the experiences of care and neurodevelopmental pathways of children with FEDs and specific NDD comorbidities.

## 1. Introduction

Feeding and eating disorders (FEDs) represent a group of psychiatric conditions characterized by the persistent disturbance of eating or eating-related behavior that results in altered consumption or absorption of food and significantly impairs physical health or psychosocial functioning. According to the fifth edition of the *Diagnostic and Statistical Manual of Mental Disorders* (DSM-5), FEDs include anorexia nervosa (AN), bulimia nervosa (BN), binge-eating disorder (BED), avoidant/restrictive food intake disorder (ARFID), pica, rumination disorder, other specified feeding and eating disorder, and unspecified feeding and eating disorder [1]. The onset and maintenance of FEDs are considered multifactorial, being sustained by multiple psychobiological agents [2], involving endocrine [3], biological [4], and psychopathological factors [5].

The treatment of FEDs is complex as these conditions are often associated with medical complications (e.g., endocrine–metabolic, gastrointestinal, cardiac, etc.) and other psychiatric comorbidities (e.g., anxiety and mood disorders, alcohol and substance use disorder, etc.) [6]. In recent decades, increasing interest has been paid to the psychopathological comorbidities of FEDs. While many studies examine psychiatric correlates in adults, evidence focusing on comorbidities of FEDs during developmental age is scarce [5,7].

Focusing on this age range, increasing interest is being paid to the possible association between FEDs and neurodevelopmental disorders (NDDs). According to the DSM-5, NDDs are defined as a group of conditions with onset in the developmental period, inducing deficits that lead to impairments of functioning. NDDs comprise intellectual disabilities (ID); communication disorders; autism spectrum disorder (ASD); attention-deficit/hyperactivity disorder (ADHD); motor disorders, specific learning disorders (SLD), and other neurodevelopmental disorders [1].

Many elements support the hypothesis of a possible link between FEDs and NDDs. especially ASD. Among them there is evidence of a familiar aggregation for these conditions, as well as of ASD-like traits in FEDs patients and the abnormal eating behaviors that characterize patients with ASD [8].

In a systematic review, Nickel and colleagues highlighted that ASD is most commonly diagnosed among patients with anorexia nervosa (AN), especially the restrictive subtype, while ADHD more frequently occurs in patients with bulimia nervosa (BN) or binge-purging AN [9]. Furthermore, the association between BN and binge-eating disorder (BED) with ADHD has been repeatedly proven [10].

Recently, an increasing number of studies investigated the presence of ASD among patients diagnosed with FEDs, although the results are controversial. Indeed, the presence of other comorbidities and the heterogeneity of the employed methods in the literature, such as different diagnostic tools and criteria, led to different results as regards the prevalence of ASD in FEDs. For instance, two studies employing parent report instruments to assess ASD among patients with AN highlighted a lower prevalence when compared with other papers [11,12]. Furthermore, the majority of the studies exclusively evaluated female patients with AN, whereas evidence assessing the presence of autistic traits in patients with FEDs other than AN is limited [13]. Our research group has extended these results by finding a correlation between autistic traits and indicators of FED psychopathology (subscales of the Eating Disorder Inventory—3 test), regardless of the body mass index (BMI) [14]. Our recent data, additionally, document an increase in the prevalence of Specific Learning Disorder (SLD) in children and adolescents with FEDs when compared to the Italian reference values [15]. When considering patients with a primary diagnosis of ASD, various eating problems, especially selective and restrictive food intake, have often been reported, resulting in insufficient caloric intake and growth failure [16].

Shan and their team approached the subject from a unique perspective by examining whether children with FEDs have a higher likelihood of developing an NDD. The research revealed a significantly greater risk of ADHD, ASD, and Intellectual Disability (ID) in children with FEDs compared to their healthy counterparts. These findings underline the need for prompt and precise monitoring of NDDs in children with FEDs, particularly in females [17]. Recently, some authors further investigated this perspective, raising the hypothesis of a possible neurodevelopmental alteration underlying all psychiatric diseases. The severity of the alteration and its interactions with other neurobiological, genetic, and environmental factors would determine multiple psychopathological trajectories, not only including NDDs such as ASD and ADHD but also schizophrenia, bipolar disorder, personality disorders, and FEDs. In particular, FEDs could be considered as a possible psychopathological trajectory of a neurodevelopmental alteration, toward which the female sex would play a role as one of many predisposing factors [8].

Despite the reported findings, suggesting a link between NDDs and FEDs, the current literature lacks studies describing the nutritional and neuropsychiatric features of children with these conditions. The present study describes, for the first time, in a limited sample, the neuropsychological, psychopathological, and nutritional features and developmental trajectories of a series of individuals with both NDDs and FEDs.

## 2. Materials and Methods

### 2.1. Study Design and Participants

The present study is a case series of 11 patients assessed between 1 January 2018 and 1 January 2023 in the regional center for FEDs during developmental age in Bologna, Italy. Inclusion criteria were (a) a diagnosis of FED, made at the enrolling center, according to the DSM-5 [1], and (b) a diagnosis of NDD, as documented by written and thorough clinical documentation. In this case series, all the families and patients provided informed consent for their data to be used for research purposes, and the hospital’s ethics process was followed throughout the conduction of this study.

### 2.2. Assessment Methods

All the patients were assessed for multiple clinical variables. These included a neuropsychological assessment using standardized tests to support the diagnosis of an NDD; a psychopathological investigation exploring the main clinical comorbidities with FED symptoms; a nutritional and anthropometric examination, providing qualitative and quantitative data on the dietary intake and modifications of body measures.

#### 2.2.1. Neuropsychological Assessment and Diagnosis of NDDs

For all the patients, the diagnosis of an NDD and a neuropsychological evaluation with the assessment of the cognitive function using standardized tests were required. These may include, preferably, a Wechsler Intelligence Scale [18] or Raven’s Standard Progressive Matrices (SPM) [19]. When further condition-specific neuropsychological NDD assessments were available (i.e., tests adopted to support a diagnosis of ASD or SLD), the results were collected and reported.

#### 2.2.2. FED Symptoms and Clinical Management

All the patients underwent a standardized diagnosis and treatment protocol for FEDs at the reference center. Their FED symptoms as well as the received treatments and outcomes were reported and discussed considering the existing comorbidities with NDDs.

#### 2.2.3. Nutritional and Anthropometric Assessment

All the patients were assessed for nutritional and anthropometric parameters at the first visit and during the clinical management. This included:

- Anthropometric measures: Baseline weight was measured by using a calibrated digital scale (Wunder WBA), without clothing on. Baseline height without shoes was measured by using a stadiometer. The weight, height, BMI, and %BMI were reported. The percentage of a normal body-mass index (BMI) for age and biological sex (%BMI) was considered instead of BMI. The use of %BMI is indicated by the report Junior MARSIPAN: Management of Really Sick Patients under 18 with AN. The percentage BMI is calculated as (BMI/median BMI for age and sex × 100) [20]. The World Health Organization BMI-for-age charts for girls and boys were used as reference values in this study [21].
- Nutritional measures: During of the first assessment, for most of the included cases, a dietitian specialized in FEDs during developmental age administered a 24 h dietary recall (24 hDR). It represents a subjective, face-to-face (or telephonic) interview [22]. The 24 hDR requires the patient to quantitatively and qualitatively describe the foods and beverages consumed in the 24 h before the interview. The types, features, quantity, preparation, brands, and dressings of foods need to be reported, together with the places of consumption and potential supplements [22]. The total caloric amount of the day has been reported. Considering the percentage of energy intake (%En) of each nutrient, it is possible to descriptively compare the percentage value of energy derived from each macronutrient (proteins, lipids, and carbohydrates) with the reference intake of nutrients and energy for the Italian population (LARN) [23].

### 2.3. Statistical Analysis

Given the small sample and the nature of the study (case series), only descriptive data were reported.

## 3. Results

### 3.1. Case Descriptions

Overall, 11 patients with NDDs and comorbid FEDs were included in the study. Their clinical features are reported in the following paragraphs and collected in Table 1. Given the high prevalence of neurological and psychiatric comorbidities in these patients, as well as the frequent occurrence of multiple NDDs in single individuals, no structured grouping for NDD profiles has been provided. Nonetheless, the list of patients follows the classification of their primary NDD diagnosis. Thus, we have included three patients with ASD (cases no. 1–3), one with ADHD (case no. 4), and seven with SLD (cases no. 5–11). Notably, the cognitive re-assessment for one of the patients with SLD (case no. 7) showed a score compatible with a diagnosis of ID.

Patient 1 is a 17-year-old boy who was diagnosed after birth with Goldenhar syndrome with left aural atresia. Bergonzini and colleagues o previously described this case [24]. As concerns his family history, his father was affected by epilepsy in his youth. The onset of FED symptoms occurred at 13 years of age, concurrently with a change in support teacher, with the occurrence of marked food and drinking selectivity (i.e., he did not take water but only other drinks). At the age of 16, he was admitted to our hospital due to severe underweight (BMI 12.7 kg/m^2^, %mBMI 62.0%) and a low food intake of about 1100 kcal/day. A diagnosis of avoidant/restrictive food intake disorder (ARFID) was made. For the evidence of poor interaction with peers and stereotyped and restricted interests, an assessment for ASD was indicated. Specific information about the early development was obtained from the caregivers and clinical documentation dating back to the the first years of life. Our evaluation was supported by the administration of the Autism Diagnostic Observation Schedule—Second Edition (ADOS-2) [25]. The obtained scores supported the diagnosis of ASD (communication: 3 points, social affect: 5 points, imagination/creativity: 2 points, restricted and repetitive behaviors: 1 point). The latest cognitive assessment (13 years) with Wechsler Intelligence Scale for Children 4th edition (WISC-IV) reported mild ID: Verbal Comprehension Index (VCI) = 70, Perceptual Reasoning Index (PRI) = 69, Working Memory Index (WMI) = 61, Processing Speed Index (PSI) = 56, Full Scale Intelligence Quotient (FSIQ) = 53, in the context of moderately limited daily social functioning. The patient then continued treatment in a residential education-integrated community specialized in the management of ASD, showing a progressive decrease in food selectivity and rigidity of thought. The patient gradually gained weight up to the current BMI of 17.6 kg/m^2^ (%BMI 82.6%).

Food selectivity is one of the features frequently associated with ASD. In the case of this patient, marked selectivity, leading to organic consequences, which represent a criterion for the diagnosis of ARFID, was the clinical element that allowed the patient’s social interactions difficulties to be re-evaluated as well and led to the suspect of ASD. Factors contributing to food selectivity in ASD include impaired sensory processing and rigidity of behavioral patterns, with difficulties with changes in routine. The presence of a second, comorbid NDD (ID), moreover, may have significantly impacted the patient’s outcome, limiting the benefits of standard, reducing the possibility of this patient directly benefiting from standard individual and group psychotherapeutic interventions to address his FED.

Patient 2 is a 17-year-old female who began experiencing symptoms of FED at the age of 15, concurrently with the national lockdown implemented during the SARS-CoV-2 pandemic. Before the onset of FED symptoms, she showed anxiety and mood disturbances. No history of FEDs, NDDs, or other mental health conditions was documented within her family. Her caloric intake was reduced up to approximately 900, alongside with excessive water consumption, accompanied by excessive water consumption of 6–7 L per day. She displayed an almost phobic avoidance of foods believed to have higher fat content. Purging behaviors, such as physical hyperactivity, self-induced vomiting, and laxative use, were implemented as means of weight loss. Over the course of one year, she reported a weight loss of 20 kg, resulting in a BMI of 17 kg/m^2^ (%BMI 82.1%). At the age of 16, she was referred to our attention following consultations with the school psychologist and the failure of brief nutritional treatment. Given her low body weight, fear of weight gain, and disturbance in body image, she was diagnosed with anorexia nervosa (AN). In addition to eating and anxious-depressive symptoms, she also exhibited self-injurious behaviors, including self-cutting. Consequently, she started a comprehensive treatment regimen comprising psychological, nutritional, and pharmacological interventions. Medications included sertraline (up to 75 mg/day) and olanzapine (up to 3.75 mg/day), and she attended a hospital day service twice a week.

Considering the presence of atypical relational features such as hypomimia, abnormal prosody, and poor understanding of nonverbal language, an assessment for ASD was performed. This assessment involved gathering information on early developmental milestones from parental interviews and early clinical records, as well as administering the ADOS-2 [25], to identify current features indicative of ASD. The evaluation revealed for the first time traits associated with ASD. Cognitive assessment using the WISC-IV scale confirmed a normal intellectual quotient (VCI = 106, PRI = 108, WMI = 94, PSI = 103, FSIQ = 105). Due to persistent purging behaviors, sertraline therapy was replaced with fluoxetine (up to 20 mg/day). Subsequently, the patient underwent two hospitalizations due to suicidal self-injurious conduct, including the use of a tight belt around the neck and ingestion of incongruous substances. Attempts were made to treat her with aripiprazole (15 mg/day) and sertraline (100 mg/day), but no significant improvement was observed in her eating and self-injurious behaviors. Currently, the patient is on fluoxetine (40 mg/day) and quetiapine (100 mg/day) therapy, which has resulted in improvements in eating dysregulation and purging behaviors. However, she continues experiencing body dissatisfaction, mood disturbances, and self-injury. Her eating behavior has shown frequent variations, transitioning from initial restriction to subsequent episodes of overeating, leading to a gradual increase in weight. Therefore, ongoing nutritional education is being provided to the patient to address restriction and binge behaviors and to stabilize her weight within the normal range (BMI 19.3 kg/m^2^; %BMI 92%). The female profile of ASD with average IQ often undergoes, as in this case, a diagnostic delay due to, among factors, better strategies of masking and relational adaptation. However, this represents a psychopathological vulnerability factor that remains untreated and can lead to mental disorders, such as FEDs. Here, the observed rigidity of thought and inadequate mentalization skills could have influenced her eating behaviors, perception of her body image, and internal parameters of self-esteem.

Patient 3 is a 14-year-old girl. In the primary school, she received specific assessments due to her learning difficulties at school reported by teachers, and a diagnosis of SLD was made (dysgraphia). No family history of FEDs, NDDs, or psychiatric disorders emerged. She came to our attention for the first time at the age of 10, with the onset of her focal epilepsy. On that occasion, specific investigations were made. Brain magnetic resonance imaging was unremarkable, and an interictal electroencephalogram (EEG) showed a focal activity in the right temporal region. A pharmacological therapy with levetiracetam was started. At the age of 13, during an outpatient visit, important restrictive nutritional aspects emerged and a first evaluation in our center for FEDs was scheduled. Due to her significant loss of weight (BMI 17.7 kg/m^2^; %BMI 91%), an outpatient care program was started and food selectivity associated with rigidly scheduled times for the meal has been reported. After collecting all data about nutritional aspects, a diagnosis of ARFID was made. For these nutritional aspects and the reported atypical social communication skills, interests, and activities, an assessment for ASD was performed. The history of early psychomotor development was collected using an Autism Diagnostic Interview-Revised (ADI-R) [26]. The presence of ASD features was clinically assessed via the ADOS-2, the Social Communication Questionnaire (SCQ) [27], and the Childhood Autism Rating Scale (CARS) [28]. The tests showed a huge discrepancy between the cognitive skills assessed, resulting in the normal range (WISC-IV: VCI = 86, PRI = 102, WMI = 97, PSI = 76, FSIQ = 88) and the immaturity of social skills and personal autonomy being indicated. A diagnosis of ASD was made. Furthermore, genetic tests including CGH-array and tests for X-fragile syndrome (with negative results) were made to exclude a syndromic framework. Her nutritional disorder could be considered an expression of her NDD, as food selectivity and pickiness greatly limited her ability to attend convivial events where meals were eaten with peers, further impairing social interaction. Co-occurrence of epilepsy may also have played a role in the onset of her FED. As long as epilepsy can be severely debilitating, impacting relationships, training at school, safety, and more, a person’s mental, emotional, and psychological well-being is also at risk. So, a FED may develop as a means of coping with overwhelming environmental triggers. Moreover, a history of epilepsy, in this case, could have directly impacted multiple neuropsychological domains, as well as the general functioning and personal autonomy, thus indirectly complicating the management of both ASD and FED symptoms.

Patient 4 is an 11-year-old boy. As regards his family history, we report that his sister was affected by NF1. At the age of 7, for distractibility and difficulties at school, he was evaluated by a neuropsychiatrist and diagnosed with ADHD. He came to our consultation at 7.5 years of age for hyperphagia with compulsive modes (marked voracity, an apparent altered sense of satiety, and snack hiding). Sometimes, he binged to the point of spontaneous vomiting. Food was described as a continuous thought for him throughout the day, with a clinical picture consistent with a diagnosis of BED. He also presented motor tics, low self-esteem, and emotional lability with restlessness and irritability. The course of his tics fluctuated with phases of increased exacerbation. Initially affecting the face, by the age of 10, they began to involve the neck and upper limbs as well. At the age of 9, he started a psychotherapeutic intervention. He never received any pharmacological therapy. At 10.5 years of age, he underwent extensive neuropsychological evaluations again, which found high cognitive resources (WISC-IV: VCI = 122, PRI = 137, WMI = 138, PSI = 118, FSIQ = 138). The skills of selective and sustained visual attention appeared sufficiently adequate; however, visual–spatial planning and verbal executive functions resulted in more impairment. His reading, comprehension, writing, and numeracy skills were within normal limits for age. Currently, his attentional times and dietary compliance have improved, but a pattern of above-normal weight and a tendency toward emotional lability, with an impact on episodes of emotional eating, remain. This patient was diagnosed with ADHD at school age. Although his level of impairment did not require pharmacological treatment, some neuropsychological features typical of ADHD, such as emotional dysregulation and impaired reward mechanisms, may have contributed to the emergence of binge eating.

Patient 5 is a 17-year-old girl. She received a diagnosis of neurofibromatosis type 1 (NF-1) at the age of 8 months and currently has an uncomplicated picture with café au lait spots, freckles, and Lisch nodules. No major psychiatric condition, FED or NDD was documented in family history. The case of this patient has been described elsewhere [29]. A psychodiagnostic evaluation was carried out for school difficulties at the age of 9, with findings of normal cognitive abilities (WISC-IV: VCI = 120, PRI = 98, WMI = 103, PSI = 103, FSIQ = 109) but errors in writing words and understanding homophonic but not homograph words, establishing the diagnosis of specific spelling disorder (ICD 10: F81.1). FED symptoms began at the age of 14, with dietary restriction and hyperactivity to lose weight. She was first evaluated six months after onset, with a weight loss of about 9 kg (BMI 17.8 kg/m^2^), and a diagnosis of AN, restrictive subtype, was proposed. In addition to eating disorder symptoms, she also presented with occasional self-injurious behaviors (self-cutting). She then started psychological and nutritional treatment and drug therapy with risperidone (0.75 mg/day) and sertraline (25 mg/day). In a few months, she had her first brief hospitalization for food refusal. One month after discharge, due to a lack of dietary compliance and continued weight loss (BMI 16.0 kg/m^2^), therapy was changed to olanzapine (3.75 mg/day) and sertraline (50 mg/day). In the following months, restrictions gradually gave way to daily binge eating followed by vomiting, requiring hospital day admission for 5 months. The disorder fell within the criteria of BN a with additional depressive traits. Therapy was changed to fluoxetine (until 60 mg/day) and aripiprazole (7.5 mg/day), with a decrease in purging. A month later, she was hospitalized for self-injurious ingestion of medication (fluoxetine), later verbalizing the need for validation of her suffering when her body showed fewer signs of the disorder. The patient is currently admitted to a specialized residence for the continuation of her FED treatment. Since NF1 may be associated with learning deficits and cognitive problems, with impairment in attention and executive functions, but also with difficulties in emotional regulation and social skills [30], as well as impaired body image and FED [29], NF-1, SLD, and FEDs could be part of a single divergent neurodevelopmental pattern in this patient.

Patient 6 is a 15-year-old girl. She was diagnosed with dyslexia and dyscalculia in primary school and obtained a support teacher. Because of this, she was bullied by her schoolmates. Her family history was unremarkable. A major event in the perception of her body image was menarche at the age of 11 with the development of secondary sexual characteristics before her other friends. The patient reported bullying for her physical appearance, for which legal action was also initiated by the family. She came to our observation at 13 years of age, on referral from her psychotherapist, for the onset of binge eating and purging behaviors (daily vomiting). Her BMI was 19.9 kg/m^2^ (%BMI 104.7%). Marked discomfort with her body and a desire to lose weight was documented as well. For these reasons, a diagnosis of BN was made. Nutritional intervention (with a diet plan and the use of oral supplements) and pharmacological intervention with fluoxetine (20 mg/day) were started. Purging behaviors decreased, but dietary restrictions to compensate for binge eating became more apparent. The patient was reassessed neuropsychologically. The level of intellectual development was found to be in the normal range. Reassessment of learning revealed reading skills in the normal range, with deficits in the area of numbers and calculating (Developmental Dyscalculia Battery, BDE2 [31]: total quotient = 54, clinical score; numerical quotient = 59, clinical score; calculus quotient < 49, clinical score). Individualized educational programming was implemented in the school setting. After a few months, a recurrence of binge eating and purging behaviors (to multiple daily episodes) was documented, so fluoxetine was increased to 30 mg/day, and nutritional management was intensified. Thereafter, an improvement in eating symptoms associated with the emergence of emotional dysregulation (outbursts of aggression toward objects) with a trend toward risky behaviors was documented. She underwent a voluntary termination of pregnancy at the age of 14. In this case, the SLD was experienced as an element of social fragility, leading to a difference among peers, which became a source of derision. This may have affected her self-esteem and self-perception, which are domains implicated in the etiopathogenesis of FEDs.

Patient 7, now a 14-year-old girl, came to our outpatient service for the first time at the age of 13. In her medical history, some learning trouble was reported, for which teachers sent her to the territorial Child Neuropsychiatry Service. After specific assessments, during her primary school attendance, a diagnosis of SLD (dysorthography and dyscalculia) with normal cognitive functioning was made. Moreover, in her family history, a brother with a congenital ophthalmological disorder was documented. The onset of the FED was at 13 years old. During the first outpatient visit, it emerged that for about two months she significantly restricted her energy intake due to her low body self-esteem. Furthermore, she began to engage in compensatory behaviors, such as self-induced vomiting two-to-three times a week. Her BMI was 25.3 kg/m^2^, and a diagnosis of atypical anorexia nervosa was made. Owing to the complexity of the situation, she started an outpatient care pathway that she is still carrying out. Moreover, to better frame her eating disorder, a revaluation of her cognitive and learning profiles was performed. Specifically, she performed the following assessment: the WISC-IV scale (VCI = 78, PRI = 80, WMI = 70, PSI = 74, FSIQ = 68), NEPSY II [32], and the Tower of London test (ToL) [33]. In conclusion, frailty in cognitive function and monitoring and planning emerged. Additionally, language processing difficulties have been confirmed. Fluoxetine was introduced when she started reporting a deflection of mood tone and progressive social withdrawal (not attending school and spending all day in her room). Furthermore, anxiety and panic attacks have been reported. In the following months, nutritional aspects became secondary to the detriment of anxious feelings about school and relationships. In this case, we could suggest that the acuity of her psychopathology may have affected the cognitive assessment. Furthermore, her learning difficulties gave a broad input in generating anxiety that lessens in dysfunctional eating behavior. Notably, the neuropsychological picture of this patient evolved over time. After a diagnosis of SLD in infancy, cognitive assessments in her adolescence documented a total IQ of 68, which, when supported with evidence of insufficient autonomies and impaired social functioning, may correspond to a diagnosis of ID [1]. Transitions and comorbidities between different NDDs in patients with FEDs may have adversely impacted the diagnosis and psychonutritional management of eating difficulties in this patient.

Patient 8 is a now a 17-year-old who came to our attention for the first time at the age of 10. No family history of mental health conditions was documented. The onset of the eating disorder is not clear because of his long history of obesity. In his medical history, it emerged that at the first grades of school, he received a learning assessment due to his difficulties at school. During the first outpatient visit, the patient had an oppositional attitude, and it emerged that he had major difficulty in weight control. His BMI was 37.1 kg/m^2^. In the following outpatient visits, the patient reported episodes of loss of control during eating, and in the following months, he continued gaining weight (until 42 kg/m^2^ of BMI), and a diagnosis of binge eating disorder was made. Although his specific learning disability has already been certified, to better define the etiology of the eating disorder presented by the patient, some cognitive and achievement assessments used to diagnose a learning disability were administered: WISC-IV scale (VCI = 74, PRI = 98, WMI = 70, PSI = 88, FSIQ = 77), Cognitive Assessment System (CAS) [34], SPM, a test for assessing Metaphor Comprehension [35], and a learning (reading, writing, and calculation) test. The intellectual functioning emerged as ascribable to a cognitive borderline level. Serious attention issues and difficulties in acquiring reading–writing and calculation procedures emerged. Fluoxetine was suggested when a depressed mood and relationship difficulties emerged, but he and his family refused the pharmacological approach. Finally, with the SARS-CoV-2 pandemic, the patient interrupted his therapeutic program. Relevantly, despite the borderline documented total IQ, a diagnosis of SLD was possible since the Italian Guidelines [36] require a case-by-case decision. Nonetheless, the evidence of low IQ scores, paired with specific learning difficulties, may limit the possibility of rigidly classifying a patient with a single NDD (SLD or borderline intellectual functioning) and should be accounted for when considering treatment plans for FEDs.

Patient 9 is a 14-year-old boy who came to our outpatient service at the age of 11 because of his selective eating behavior that determined a significant loss of weight (BMI 13.0 kg/m^2^). His mental health and family history were unremarkable. In his medical history, it emerged that in the first grades of school, due to his learning difficulties reported by teachers, he received achievement tests used to determine his academic skill and knowledge in specific areas, confirming the diagnosis of SLD. Furthermore, a chronic motor tic disorder has been reported since he was 10. During subsequent visits, it emerged that he had a significant disinterest in food associated with anxious thoughts and social inhibition. Considering the dysfunctional eating attitude and his very low weight, a diagnosis of ARFID was made. Owing to the clinical complexity, a diagnostic revaluation was made with non-verbal tests (SPM) and projective tests. The non-verbal test confirmed good logical-deductive skills while projective tests reveal insecurity and difficulties in interpersonal relationships. In the following year, he improved his energy intake, especially with snacks and dietary supplementation, and he started a therapeutic path for his relational issues with the territorial Child Neuropsychiatry Service. With this global take-over, his motor tics also improved.

Patient 10 is a 16-year-old boy who came to our attention for the first time at the age of 13. In his family history, it emerged that his mother had been diagnosed with AN whilst he was very young, and his medical history and SLD (dyslexia and dyscalculia) were diagnosed in the first grades of school. The onset of his FED symptoms dates back to the pandemic when he started having binge episodes between main meals and frequent snacks in the afternoon hours which he spent at home alone, to such an extent that in a few months, he gained 20 kg. Furthermore, during the first interview, a feeling of self-disgust, a depressed mood, and relational difficulties with agoraphobic thoughts were revealed. Due to the global clinical situation, he started an outpatient program, and fluoxetine was introduced. His BMI was 36.8 kg/m^2^ and a diagnosis of binge eating disorder was made. In the following months, outpatient nutrition education was started, which led to a reduction in weight of 11 kg in 6 months (BMI 31.9 kg/m^2^) with a simultaneous increase in height. After starting psychotherapy, he verbally expressed suicidal ideation with an improvement in mood. At the age of 15 years old, due to his anxious thoughts about school and a sense of constant insecurity, a revaluation of his cognitive and learning skills was performed. He underwent the following assessments: SPM, a test for the reception of grammar (TROG-2), a Neuropsychological Assessment Battery, and a learning (reading, writing, and calculation) test. His cognitive profile was documented, while the patient showed frailty in fast reading, graphomotor writing skills, and the computing area. In the same period, the patient stopped exercising, reporting that he felt disadvantaged in frequenting crowded environments such as the gym. Binge episodes, conversely, increased in frequency, leading to new weight gain (BMI 36.7 kg/m^2^). In this case, we could suggest that learning frailties could have contributed to psychopathology, resulting in uncontrolled eating behavior.

Patient 11 is a 15-year-old girl. She was diagnosed with epilepsy with absence seizures at the age of 5. In her family history, a case of epilepsy in the maternal line was reported. Her seizures were drug-resistant and were treated with valproic acid and ethosuximide in monotherapy and levetiracetam, lamotrigine, zonisamide, and topiramate in polytherapy with ethosuximide or even clobazam. After one cycle of ACTH, therapy with valproate and ethosuximide in combination provided good control of the episodes. At the age of 7, she underwent a neuropsychological evaluation with findings of an intellectual level in the normal range (SPM) resulting at the 57th percentile; a WISC-IV showed the following scores: VCI = 94 and PSI = 97, with adequate attentional, visuoperceptual, and visuomotor integration skills. The tests administered showed, however, a specific learning disorder of reading–writing. The patient was then assisted in school learning by a support teacher. Due to clinical remission, the antiepileptic therapy was stopped at 9 years of age. She came to our observation at 11 years of age for marked food selectivity and low weight with a BMI of 13.3 kg/m^2^ (%BMI 76.4%). She was eating three meals and three snacks a day, preferring sweet and savory snacks, for a total of about 1200 kcal/day, a low-calorie diet for that age, hyperlipidic and low in fiber. She also presented with recurrent abdominal pain, so organic causes were ruled out. A diagnosis of ARFID was made. Oppositional behaviors, low frustration tolerance, emotional lability with easy crying, and outbursts of anger with verbal and physical hetero-aggression toward family members were also reported. Nutritional treatment (with a diet plan and oral supplements) and individual and family psychotherapy were therefore undertaken. Drug therapy with fluoxetine was also proposed but refused by the family. At 13.5 years of age, her food selectivity, thymic tone, and abdominal pain symptoms had improved, with a BMI of 14.8 kg/m^2^ (%BMI 77.5%). At 14 years of age, the patient had her menarche, with a BMI of 15.6 kg/m^2^ (%BMI 79.6%). One month later, following a traumatic bone fracture and subsequent infectious complication, the patient implemented dietary restrictions, avoiding some meals. These behaviors were associated with mood deflection and reversal of the sleep–wake rhythm with night meals. A clear desire to lose weight was not reported, nor was body image disturbance. In the following months, she experienced the return of more regular meals and mood. Currently, the patient has a BMI of 15.9 kg/m^2^ (%BMI 77.9%).

This patient underwent cognitive and learning assessment since she had drug-resistant epilepsy, which could impair these areas. SLD, therefore, is diagnosed before the onset of feeding symptoms. Her long medical history, with hospitalizations and several pharmacological interventions, may have created a trait of vulnerability that led to impairment in emotional regulation skills, with oppositional and refusal behaviors, until the onset of the FED.

### 3.2. Nutritional Assessment Together with Demographics and Anthropometric Parameters

The demographic, anthropometric, and nutritional parameters of each patient are shown in Table 2. The anthropometric parameters were measured at the first ambulatory access and subsequent outpatient check-ups. Based on the data collected, the %BMI indicated by the Junior MARSIPAN report [20] was calculated. 

The nutritional assessment was carried out using the 24 hDR. For two patients (patients 4 and 8), the food investigation was not reported in the medical record.

The median %En for carbohydrates was closer to the lower limit of the recommendations (45–60%) for patients 3, 5, 6, and 7, as the dietary composition was mainly high protein, high-fat food. All patients were offered nutrition education (NE), which can be defined as any set of learning experiences designed to encourage the voluntary adoption of eating and other nutrition-related behaviors conducive to well-being and health.

## 4. Discussion

Our case series provided the neuropsychological, neuropsychiatric, and nutritional data of 11 patients with NDDs and comorbid FEDs.

A summary of the diagnoses associated with our sample is reported in Table 1. Our cases are clinically heterogeneous, and for this reason, it is difficult to draw an inherent conclusion between the association of FEDs and NDDs. Complicating matters, there is an association between different NDDs in the same cases (see case numbers 1, 3, 4, 7, 8, and 9) and epilepsy (see case numbers 3 and 11), and rare genetic syndromes (case number 1). What is important to highlight is (a) the time of diagnosis of NDDs and FEDs; (b) the type of FED associated with the type of NDD and others; (c) the prognosis tied up of this association; and finally, d) the treatment.

Clinicians should note that NDDs frequently occur in comorbidity, making it difficult to assign a rigid diagnosis to a single individual. The presence of multiple NDDs in a developmental-age subject and during its lifespan can lead to a complex clinical presentation and make diagnosis and treatment challenging.

The diagnosis of NDDs has always preceded that of FEDs, except in cases of ASD. In these cases, the particular features of the symptoms that emerged with FED likely led to a re-evaluation of other domains, such as social skills, type of interests, and quality of communication. In case number 2, for example, it is important to remember the difficulty of making a diagnosis of ASD in females. Early detection of feeding disorders in children with ASD is essential, as delays in diagnosis and treatment can lead to long-term complications such as malnutrition and poor physical health [37]. ASD showed a direct impact on the clinical features of FEDs (AN for one case; ARFID for two cases). Typically, ASD symptoms hindered the possibility of active participation in standard treatments for FEDs in our patients. ASD has been shown to have a significant impact on the treatment of children and adolescents with ARFID and or/AN.

Different types of FEDs are reported associated with NDDs (see Table 1), and the limited number of cases prevents clinicians from concluding. Regardless, we can affirm that individuals with ASD have a higher likelihood of experiencing ARFID and AN compared to the general population [38]. As recently documented, ARFID in young children may represent an early sign of ASD [39]. Individuals with AN and comorbid ASD may have a poorer response to standard treatments [40]. This may be due to the challenges in treating both conditions simultaneously, as the neurobiological and psychological mechanisms underlying AN and ASD are complex and interconnected [41]. We may hypothesize that the existing link between ASD and FEDs is based on multiple factors. Hyper- or hyporeactivity to sensory input or unusual interest in sensory targets, as well as insisting on sameness, adhering inflexibly to routines, and abnormalities in the intensity and focus of interest may alter feeding patterns across all ages for individuals with ASD [37,42,43]. Cognitive and interpersonal factors, moreover, including altered theory of mind, empathy, and executive functions, may be responsible for similarities and comorbidities between ASD and AN in adolescence [38,44,45,46].

Only one of the included patients presented with ADHD. This condition occurred in comorbidity with a BED. Notably, motor tics and emotional liability co-occurred, suggesting multiple impairments in neurodevelopmental pathways. These conditions impacted the eating pattern of the individual, with the evidence of episodes of emotional eating, and a poor response to FED treatments. The co-occurrence of ADHD and eating disorders, particularly binge eating disorder (BED), has been frequently reported in the literature. Individuals with ADHD and BED have been found to experience more severe eating disorder symptoms, including binge eating frequency and weight gain [47] Moreover, ADHD symptoms such as impulsivity and emotional dysregulation may also be linked to binge eating behaviors [48]. There are several possible mechanisms responsible for the comorbidity between ADHD and FEDs. One potential mechanism is related to shared neurobiological underpinnings between these conditions, including alterations in the dopamine system and prefrontal cortex function, which may contribute to both ADHD symptoms and FED behaviors [49,50]. Additionally, impulsivity and emotional dysregulation, which are core features of ADHD, may contribute to binge eating and other FED symptoms [10,51]. Other factors, such as genetics, environmental factors, and individual differences in temperament and personality, may also contribute to the co-occurrence of these conditions.

ADHD may also affect treatment outcomes in individuals with eating disorders, and the potential impact of ADHD treatments on patients with BED may be hypothesized [52]. Early detection and treatment of ADHD symptoms may improve eating disorder outcomes in individuals with comorbid ADHD and BED [47]. Thus, further research should assess whether, in addition to standard treatment approaches, such as cognitive–behavioral therapy, interventions aimed at improving ADHD symptoms, such as behavioral interventions and pharmacotherapy, may be beneficial in improving overall outcomes.

Seven of the included patients presented with an ID or SLD and a comorbid FED. This varied across multiple DSM-5 diagnosed FEDs (AN, AAN, BN, BED, and ARFID). In most of these patients, learning difficulties were expressed as an impairment in achieving basic school results and could have altered the self-efficacy and self-esteem of the affected individuals. Despite the difficulty of establishing a direct causal relationship between SLD and the development of a FED, potential links between these conditions may exist. A higher prevalence of psychiatric and neurodevelopmental diseases has been found in Swedish patients with reading difficulties, with a similar risk in their siblings, according to large-scale cohort research [53]. In clinical samples, our research group assessed the documentation of 262 children and adolescents with FEDs [15]. Overall, 25 patients out of 262 (9.54%) showed a comorbid diagnosis of SLD. This SLD prevalence was higher than the Italian reference data (4.9% in the school year 2018/19). Males presented more frequent comorbidity with SLD, but no diagnosis of SLD was associated with any specific FED. Relevantly, one of the involved patients with SLD presented with NF-1, a condition frequently associated with SLD and characterized by specific neuroradiological and neuropsychological features [54]. We may hypothesize that individuals with ID/SLD may face challenges in understanding and processing information about food and nutrition, leading to difficulties with meal planning and preparation. Additionally, some may have sensory processing difficulties that impact their food choices and intake. These challenges, coupled with social and emotional factors, could increase the risk of developing FEDs in this population. Further research should investigate whether specific neuropsychological deficits underlying an ID/SLD may be associated with greater severity or different FED symptoms.

Finally, it should be noted that two of the included patients with an NDD and a FED also presented comorbid epilepsy. Both patients had SLD, and one of them also had ASD. They were both diagnosed with ARFID. Currently, in the literature, few studies have sought a possible connection between FEDs and epilepsy, while more clinical studies have been carried out to analyze the relationship between NDDs and epilepsy. Notably, Kolstad and colleagues observed a greater prevalence of feeding symptoms among adolescents with epilepsy in a Norwegian cohort. Data from a questionnaire for 19,995 participants, including 247 with epilepsy, showed that adolescents with epilepsy had a higher risk of FEDs, unhealthy eating, and less satisfaction with their appearance. Male adolescents with epilepsy had a higher risk of dieting (OR 3.1) and less satisfaction with their appearance (OR 0.4), while females with epilepsy had an increased risk of unhealthy eating [55]. The influence of antiepileptic therapy on the risk of developing symptoms of altered feeding could be due to both metabolic and psychotropic effects [56,57].

Indeed, it is known that learning and behavioral disorders are more common in people with epilepsy than in the general population. To explain this, multiple factors may have been associated, such as various psychosocial, medication, and epilepsy-related factors [58]. Instead, about ASD, Keller reported that roughly 5–46% of individuals with ASD also suffer from epilepsy and approximately 30% of children with epilepsy meet the criteria for ASD [59,60]. Such an increased prevalence suggests that these two conditions, ASD, and epilepsy, might share a common pathophysiological basis.

Despite having a small sample size, this study represents the first work to thoroughly assess these three main clinical domains in this population. NDDs may occur as a comorbidity in severe psychiatric conditions [61], including FEDs, with a potential impact on the course and outcome of treatment. For example, individuals with ASD and eating disorders may require modifications to standard treatment approaches [62]. As frequently documented in child psychiatry, both FEDs and NDDs, moreover, share a complex pathogenesis, which involves perinatal, biological, and social factors [4,63].

The patients reported in this case series highlight the difficult developmental and treatment pathways for children and adolescents experiencing comorbidity between FEDs and NDDs. Given the increasing evidence for treatment interventions aimed to improve symptoms and quality of life in people with NDDs [64], the early recognition of these conditions could have a direct impact on the management of the involved patients.

Further research is needed to identify effective treatments for individuals with comorbid NDDs and feeding disorders as well as to understand the underlying mechanisms of these conditions.

### Limitations

This study has some limitations. The small sample and the case series nature limit the generalizability of the results. Additionally, multiple, different diagnoses included here, both for FEDs and NDDs, hinder the possibility of drawing disease-specific conclusions. The included patients, finally, received multiple and different assessments, related to their individual NDDs, thus avoiding establishing clear correlations. Nonetheless, this study also shows some strengths. It is the first study to present a multiaxial assessment for individuals with FEDs and comorbid NDDs, providing data on the psychopathological and nutritional features of these uncommon, related conditions. The nature of the study center, a third-level hospital center for FEDs, specialized in the diagnosis and management of NDDs, supported this point. Moreover, the case series nature permits the reporting of individual developmental and eating pathways, with the possibility of identifying relevant changes at multiple time points.

## 5. Conclusions

The association between FEDs and NDDs represents an important clinical condition that requires attention and precocious diagnosis for treatment and prognostic consequences. This case series, albeit numerically limited, represents, to the best of our knowledge, the first representation of this association to systematically assess the neuropsychological, psychopathological, and nutritional features of a sample of children and adolescents with FEDs and comorbid NDDs or vice versa. The patients included in this paper presented specific developmental patterns, with evidence suggesting, despite not proving, a potential link between these two conditions. Clinicians and researchers need to possess diagnostic skills for both pathological conditions. Future research should focus on exploring the association between specific types of FEDs and comorbid NDDs. For ASD, investigating the difference in neuropsychological features between individuals with comorbid ARFID and comorbid AN would provide valuable insights. Understanding the potential benefits of incorporating behavioral interventions and pharmacotherapy for ADHD and in the clinical expression of BED may be essential. Examining the relationship between SLD and different FEDs, such as BN or ARFID, particularly in the field of executive functions, could provide valuable information. Additionally, studying the influence of epilepsy and its treatment implications on ID and comorbid FEDs would contribute to better understanding and management. By focusing on specific FEDs profiles and their relationships with NDDs, future research can identify tailored interventions and shed light on the underlying mechanisms involved.

## Figures and Tables

**Table 1:** Patients subtests results of the cognitive assessment WISC-IV and SPM.

Patients	FED	NDD	Comorbidities	VCI	PRI	WMI	PSI	FSIQ	SPM
Patient 1	ARFID	ASD	Goldenhar syndrome, ID	70	69	61	56	53	-
Patient 2	AN	ASD	-	106	108	94	103	105	-
Patient 3	ARFID	ASD	SLD/Epilepsy	86	102	97	76	88	-
Patient 4	BED	ADHD	Tic	122	137	138	118	138	-
Patient 5	AN/BN	SLD	NF-1	120	98	103	103	109	-
Patient 6	BN	SLD	-	-	-	-	-	-	-
Patient 7	AAN	SLD/ID	-	78	80	-	74	68	-
Patient 8	BED	SLD/BIF	-	74	98	70	88	77	32 pct
Patient 9	ARFID	SLD	Tic	-	-	-	-	-	63 pct
Patient 10	BED	SLD	-	-	-	-	-	-	57 pct
Patient 11	ARFID	SLD	Epilepsy	94	-	-	97	-	-

Abbreviations: AAN = Atypical Anorexia Nervosa, ADHD = Attention-Deficit/Hyperactivity Disorder, AN = Anorexia Nervosa, ARFID = Avoidant/Restrictive Food Intake Disorder, ASD = Autism Spectrum Disorder, BED = Binge Eating Disorder, BIF = Borderline Intellectual Functioning, BN = Bulimia Nervosa, FEDs = Feeding and Eating Disorders, FSIQ = Full Scale Intelligence Quotient, NDD = Neurodevelopmental Disorder, NF-1 = Neurofibromatosis type 1, PRI = Perceptual Reasoning Index, PSI = Processing Speeding Index, SPM = Standard Progressive Matrices, UFED = Unspecified Feeding and Eating Disorder, SLD = Specific Learning Disorder, VCI = Verbal Comprehension Index, WISC-IV = Wechsler Intelligence Scale for Children 4th edition, WMI = Working Memory Index.

**Table 2:** Demographics, family history, and anthropometric and nutritional features of the patients.

Patients	FED	Family History	Age	Sex	BMI (kg/m^2^)	%BMI	Energy Intake (kcal)	Proteins %En	Lipids %En	Glucides %En	Nutritional Treatment
Patient 1	ARFID	Epilepsy (father)	16	M	12.7	62.0%	1058	15.9%	27.2%	57.8%	NE
Patient 2	AN	/	15	F	17	82.1%	987	23.5%	25.5%	51.1%	NE + Diet.
Patient 3	ARFID	/	13	F	17.7	91.2%	1139	23.5%	38.7%	38.3%	NE
Patient 4	BED	NF-1 (sister)	7	M	21.2	135.0%	/	/	/	/	NE
Patient 5	AN/BN	/	15	F	17.8	88.1%	1323	25.1%	32.0%	42.3%	NE
Patient 6	BN	/	13	F	19.9	104.7%	828	27.1%	32.6%	41.1%	NE
Patient 7	AAN	Congenital ophthalmological dis. (brother)	13	F	25.3	132.5%	1226	18.6%	40.4%	40.4%	NE
Patient 8	BED	/	10	M	37.1	222.2%	/	/	/	/	NE
Patient 9	ARFID	/	11	M	13	76.5%	1425	16.8%	31.6%	53.3%	NE + Diet.
Patient 10	BED	AN (mother)	12	M	36.8	186.8%	2234	19.3%	27.8%	52.8%	NE
Patient 11	ARFID	Epilepsy (maternal line)	11	F	14.3	82.2%	1283	17.5%	32.3%	52.1%	NE

Abbreviations: FEDs = Feeding and Eating Disorders, BMI = Body Mass Index, %En: percentage of Energy Intake, ARFID = Avoidant/Restrictive Food Intake Disorder, UFED = Unspecified Feeding and Eating Disorder, AN = Anorexia Nervosa, BED = Binge Eating Disorder, BN = Bulimia Nervosa, AAN = Atypical Anorexia Nervosa, NE = Nutritional Education; NF-1 = Neurofibromatosis, type 1.

## Data Availability

The data assessed and reported here can be obtained from the authors upon reasonable request and following ethical and privacy principles.
